# Web-Based Research Trends on Child and Adolescent Cancer Survivors Over the Last 5 Years: Text Network Analysis and Topic Modeling Study

**DOI:** 10.2196/32309

**Published:** 2022-02-01

**Authors:** Hyun-Yong Kim, Kyung-Ah Kang, Suk-Jung Han, Jiyoung Chun

**Affiliations:** 1 Logos Health Design Institute Sahmyook University Seoul Republic of Korea; 2 College of Nursing Sahmyook University Seoul Republic of Korea

**Keywords:** text network analysis, topic modeling, cancer survivors, child, adolescent, research trends, knowledge structures

## Abstract

**Background:**

Being diagnosed with cancer during childhood or adolescence can disrupt important periods in an individual’s physical, psychosocial, and spiritual development and potentially reduce the quality of life (QOL) after treatment. Research is urgently required to improve the QOL for child and adolescent cancer survivors, and it is necessary to analyze the trends in prior research reported in international academic journals to identify knowledge structures.

**Objective:**

This study aims to identify the main keywords based on network centrality, subgroups (clusters) of keyword networks by using a cohesion analysis method, and the main theme of child and adolescent cancer survivor–related research abstracts through topic modeling. This study also aims to label the subgroups by comparing the results of the cohesion and topic modeling.

**Methods:**

A text network analysis method and topic modeling were used to explore the main trends in child and adolescent cancer survivor research by structuring a network of keyword (semantic morphemes) co-occurrence in the abstracts of articles published in 5 major web-based databases from 2016 to 2020. A total of 1677 child and adolescent cancer survivor–related studies were used for data analyses. Data selection, processing, and analyses were also conducted.

**Results:**

The top 5 keywords in terms of degree and eigenvector centrality were *risk*, *control interval*, *radiation*, *childhood cancer treatment*, and *diagnosis*. Of the 1677 studies used for data analyses, cluster 1 included 780 (46.51%) documents under *risk management*, cluster 2 contained 557 (33.21%) articles under *health-related QOL and supportive care*, and cluster 3 consisted of 340 (20.27%) studies under *cancer treatment* and *complications*.

**Conclusions:**

This study is significant in that it confirms the knowledge structure based on the main keywords and cross-disciplinary trends in child and adolescent cancer survivor research published in the last 5 years worldwide. The primary goal of child and adolescent cancer survivor research is to prevent and manage the various aspects of the problems encountered during the transition to a normal life and to improve the overall QOL. To this end, it is necessary to further revitalize the study of the multidisciplinary team approach for the promotion of age-specific health behaviors and the development of intervention strategies with increased feasibility for child and adolescent cancer survivors.

## Introduction

### Background

More than 300,000 children worldwide are diagnosed with cancer every year; every 3 minutes, a family somewhere in the world receives the unexpected and shattering news that their child has been diagnosed with cancer [1]. According to a 2017 survey of major causes of death across all races and sexes in the United States, malignant neoplasm was the leading cause of disease-related death in children and adolescents [2]. Approximately 137 million children are predicted to be diagnosed with cancer worldwide between 2020 and 2050 [3].

With significant advances in diagnosis and increase in multimodal treatment, childhood cancer survival rates have improved significantly in recent decades [4]. In the case of 5-year survivors of childhood cancer, the 30-year cumulative survival rate is 81.9% (95% CI 81.1-82.7) [5]. However, most child and adolescent cancer survivors experience additional problems and health effects after cancer treatment. More than 60% of survivors report with at least one chronic disease, and more than one-third report experiencing a reduced quality of life (QOL) with at least 2 complications [6,7].

Late complications experienced by child and adolescent cancer survivors may be psychosocial and behavioral, such as depression, anxiety, and risky health behaviors, or physical, such as cardiovascular disease, secondary cancer, and hormonal and immune deficiencies [8,9]. Even after full recovery, many child and adolescent cancer survivors have a high risk of death and morbidity resulting from secondary infections [10,11]. In other words, being diagnosed with cancer at this age can disrupt key periods of physical, social, and psychological development and potentially reduce QOL after treatment [12,13]. Therefore, significant effort is required to improve the QOL of child and adolescent cancer survivors.

More targeted research is needed to improve the QOL of child and adolescent cancer survivors; it is necessary to analyze previous research and organize the research results. A systematic literature review and meta-analysis, which are common methods used to intensively analyze intervention effects in the field of interest, are based on specific research questions and analyzed for a limited number of relevant papers [14]. Although it is possible to investigate the outcome of a topic of interest in this way, there is a limitation in that the overall knowledge structure of the context in which the major research concept and topic are studied or the type of connections between the main concepts cannot be known.

Exploiting a text network analysis to study research trends provides useful information with new ideas for future research [15,16]. These results can be used to form a body of knowledge by quantitatively analyzing a large number of papers and qualitatively interpreting the discovered knowledge structure to identify a certain pattern in the scattered data [17]. In addition, because numerous related papers are collected and used for analysis, this method has a significant quantitative scientific basis in terms of the research results and in identifying core research topics in academic fields and its relevance to various subtopics. In particular, it is possible to intuitively explore the knowledge structure by visualizing such research results [18,19].

Through text network analysis, it is possible to check the connectivity (links) between the words included in the documents (in our case, the words in the abstracts) and the connectivity group (community) of keywords based on their links. In addition, if a topic modeling analysis, which has the advantage of identifying topics representing the community based on the probability distribution of words included in the document, is applied simultaneously within a text network analysis, then trends based on the core topic of research in the field of interest can be clearly identified. Although the probability distribution of words in a document does not have any intuitive meaning, researchers can interpret the meaning of a specific topic, and thus, the extracted topic can be used as important information representing the document [20,21].

In the field of adult cancer and cancer survivor care, the number of studies applying a text network analysis to confirm the knowledge structure and overall research trends in the field of interest have increased over the past 10 years [16,22-25]. However, few studies that were published in 2017 applied a network analysis method to child and adolescent cancer survivors [15]. Because information technology has been undergoing rapid advancements, it is important that research on child and adolescent cancer survivors is shared all over the world.

To improve the QOL of child and adolescent cancer survivors based on the latest information, it is necessary to comprehensively identify research trends related to child and adolescent cancer survivors using text network analysis on studies published in internationally recognized academic databases. Therefore, in this study, using a text network analysis method and topic modeling, the main research concepts of previous studies on child and adolescent cancer survivors published in international academic journals in the last 5 years were reviewed, and research trends were identified.

### Objectives

The specific goals of this study that analyze the abstracts of child and adolescent cancer survivor–related research papers are as follows: identifying the main keywords based on network centrality, finding the subgroups of a network of keywords using a cohesion analysis, clustering the main theme of child and adolescent cancer survivor–related research abstracts through topic modeling, and comparing and analyzing the results of cohesion and topic modeling.

## Methods

### Study Design

**Figure 1:**
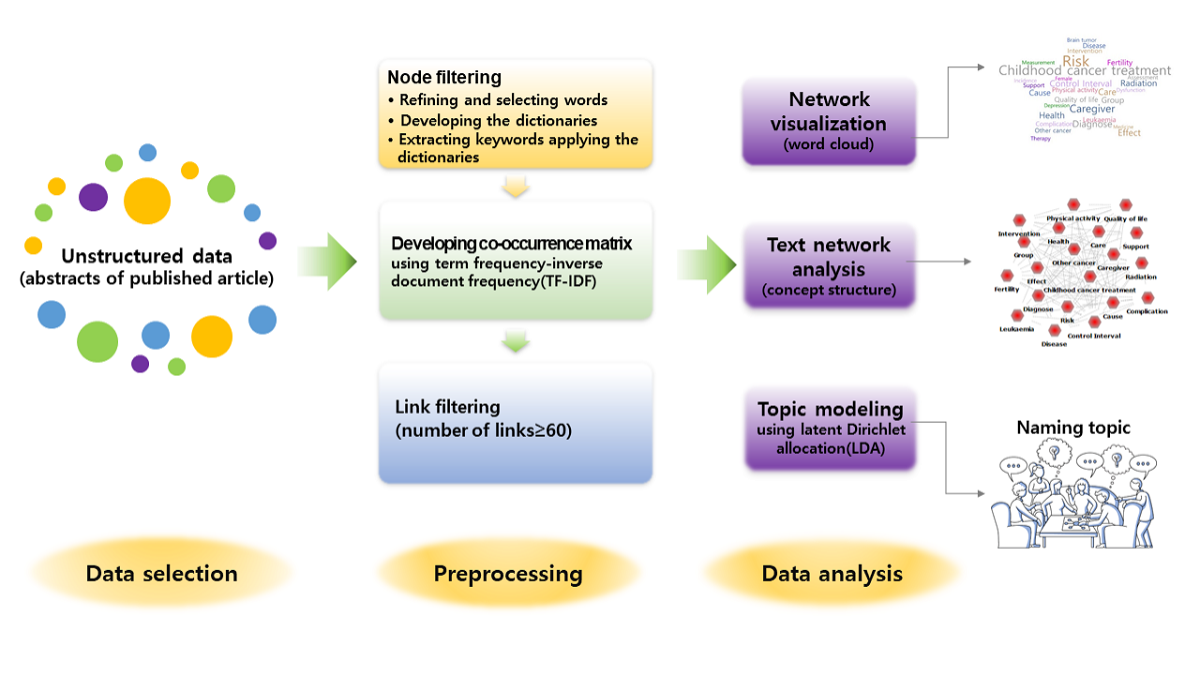
The process of this study.

This study used text network analysis and topic modeling to explore the main research trends of child and adolescent cancer survivors by structuring a word co-occurrence network among the keywords (semantic morphemes) in the abstracts of articles published in 5 major web-based databases between 2016 and 2020. The process used is illustrated in Figure 1.

### Ethical Considerations

This study was approved by the Institutional Review Board of Sahmyook University (IRB No. 2020116HR). The data were collected from published articles in web-based databases, and no harm or risk was posed by the research to the participants. In addition, the collected data will not be used for any purpose other than for this study.

### Data Selection

#### Keyword Selection

**Figure 2:**
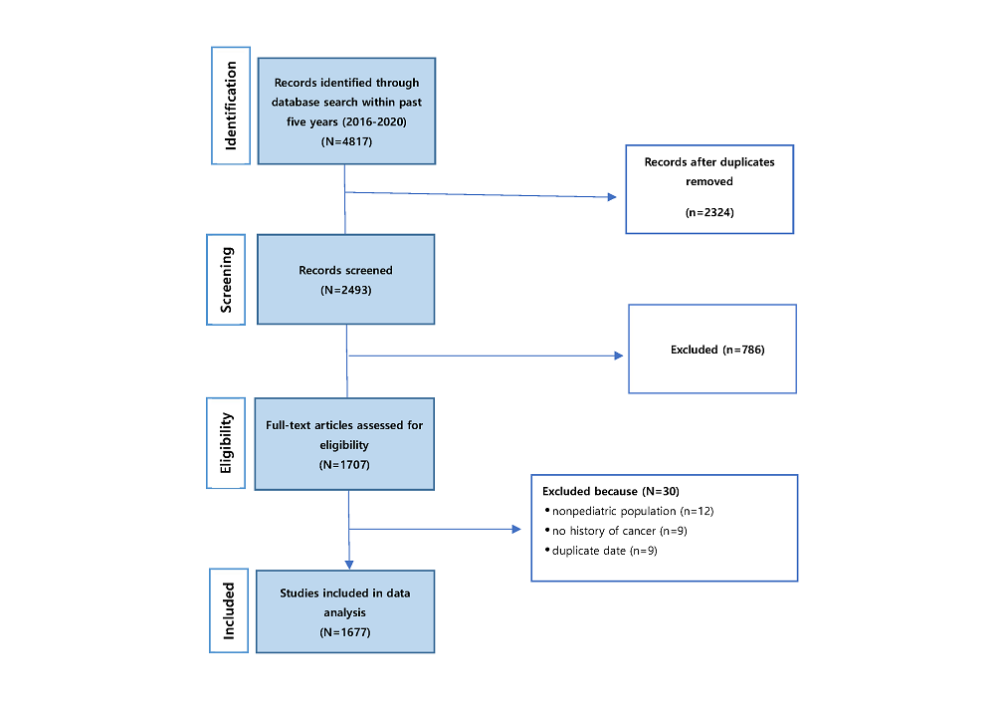
A PRISMA (Preferred Reporting Items for Systematic Reviews and Meta-Analyses) flow diagram.

The first search to retrieve published articles from all time periods in 5 major databases (PubMed, CINAHL, Cochrane, Embase, and PsycInfo) was conducted in January 2021. The main keywords used included *Adolescent* [Mesh] OR *Child* [Mesh] OR *Pediatrics* [Mesh] and *Cancer Survivors* [Mesh] along with *Childhood cancer survivors*, *Adolescent cancer survivors*, and *Pediatric cancer survivors*. Only articles written in English during the 5-year period of 2016-2020 were selected. The article identification and selection process used the PRISMA (Preferred Reporting Items for Systematic Reviews and Meta-Analyses) flow diagram protocol (Figure 2) to retrieve 4817 articles during the initial search; 51.75% (2493/4817) articles were retained after eliminating duplicates (2324/4817, 48.25%). Each title was reviewed in accordance with this study’s topic of interest, and 31.52% (786/2493) irrelevant studies were excluded. In all, 68.47% (1707/2493) of full-text articles were assessed for eligibility. Of the 1707 articles assessed, 30 (1.76%) that included a nonpediatric population (12/30, 40%), no history of cancer (9/30, 30%), and duplicate data (9/30, 30%) were excluded. Finally, 67.27% (1677/2493) abstracts were collected after reviewing their adequacy, and a network analysis was conducted on the data.

#### Collection of Abstracts

A total of 2 researchers (HYK and KAK) of this study reviewed the titles of the retrieved data to identify the criteria for selection and exclusion. Moreover, 2 other researchers (SJH and JC) of this study repeated the former review process. A total of 1677 studies were selected for the final data analyses.

### Preprocessing

#### Overview

The final selected abstracts were categorized to generate compatible unstructured data that could be accessed by NetMiner (Cyram) version 4.0. NetMiner is a validated program that conducts text network analysis by extracting a keyword from the abstracts and applying automatic filtration of the parts of speech (eg, nouns, verbs, and adjectives), generating a word co-occurrence matrix, and visualizing the analysis results [26].

First, to create compatible unstructured data, we categorized the retrieved studies based on publication year, author, title, abstract, and keywords using Microsoft Excel 2016. The raw data were then imported into NetMiner, and 10,786 words were identified as the main set of nodes.

#### Node Filtering

##### Word Refining and Selection

In all, 2 researchers of this study conducted a brief review of the first main node set of 10,786 words, regardless of the occurrence frequency. There is no scientific protocol available for the cut-off standard; however, it is widely accepted that words that only appear once can be excluded. During the review process, most of the words with a frequency of 1-4 showed inaccurate spellings or errors, irrelevance with respect to the main concept of the study, and general verbs or adjectives that were considered far removed from the actual content. Therefore, both researchers reached a consensus to exclude words with a frequency of <5, thus leaving 2755 words as a result.

##### Development of Dictionaries

For meaningful text network analysis, refining the semantic morphemes is important [17,26]. For the further filtering process, 4 researchers conducted a detailed review of each term and created a filtering dictionary. A total of 3 types of filtering dictionaries were created during this review. First, the thesaurus dictionary contained the identification of keywords that had the same definition but were listed in different ways (eg, *QOL*, *quality of life*, and *Quality of Life*). An exclusion dictionary was created to eliminate words that were not significantly related to the study content (eg, *apple*, *Netherlands*, and *cars*), and terms that were composed of multiple words representing a single definition (eg, *physical activity* as a single term, not *physical* and *activity* separately) were listed in the defined dictionary.

##### Keywords Extracted by Applying Dictionaries

The dictionaries were applied using the NetMiner software while importing unstructured data. With a network structure in which people in a social network analysis are nodes (points) and relationships among people are links (lines), a text network analysis helps us understand the relationship among words comprehensively and intuitively by providing a sociogram in which words are applied as nodes, and co-occurrence relationships are applied as links [18]. This filtering process was repeated until the main node set of words was clearly noticeable without any errors (eg, uncapitalized terms, undefined terms, or unorganized synonyms). When the first filtering process was completed, 217 words remained in the main node set.

#### Development of Co-occurrence Matrix for Keywords (From 2-Mode to 1-Mode Network)

In this study, an abstract was considered a single document unit; each term represented a single node unit, and the initial word-document network was created (2-mode). Then, to process the comembership between terms, a co-occurrence word network (1-mode) was generated using the recoded weight value of term frequency-inverse document frequency (TF-IDF). The TF-IDF value reflects both the importance and frequency of a term occurring in a set of documents; therefore, it can be considered as an appropriate value for conducting a keyword network analysis [17,18,26]. An inner-product-proximity measure was adopted during the mode-conversion process.

#### Link Filtering

The generated 1-mode network consisted of 10,826 links. An excessive number of links in a network must be filtered to clearly view the results for network visualization. The cut-off standard for link reduction was determined by conducting a link-reduction simulation. A link frequency of ≥60 was chosen for the reduction, and 378 links were finally included in the data analysis.

### Data Analysis

NetMiner version 4.0. was used for data analyses.

#### Network Visualization

Network visualization is the styling of nodes and links such that the connecting structure can be intuitively expressed [17,20]. A total of 3 simulations (with a link frequency of ≥55, ≥60, and ≥65) were conducted to visualize the map of the 1-mode network, and a consensus among the authors was reached to adopt a link frequency of ≥60, which best reflects the major trends in childhood cancer research. We applied a word cloud to visualize the network.

#### Text Network Analysis

Centrality is a useful index employed to determine the node (keyword) that lies at the center of the network, and the rank is determined based on its relativity rather than the absolute size. A node with a high centrality value can be considered as the core-acting node in a network [17,18,27]. This study adopted the following two approaches: degree and eigenvector centrality.

Degree centrality measures the number of connections carried by a single node, which indicates that a node with a higher degree centrality value and a higher number of connections within a network can be determined as a core keyword. The concept of eigenvector centrality is somewhat similar; however, rather than determining the direct connections of a node, it overviews the entire network to discover the most influential keyword by counting its surrounding neighbors. The standardized value of centrality lies between 0 and 1 and is used to determine the core-acting keywords in the theme of this study [17,26].

A cohesion analysis was conducted to identify the subtopic groups and the division of communities belonging to the co-occurrence word network [17,28]. In the first step, the giant component was extracted, and a community analysis was conducted. The largest modularity (best cut) is regarded as the most optimized value, with values of 1.25-2.75 indicating normal values and 2.75-3.5 indicating good values [17,28]. A higher modularity with a positive value means that the community is significantly divided; this link density within the group is high and between groups is low [17,18,28].

When visualizing the results of the network analysis, it is difficult to interpret the connections between many nodes and links because of their complexity. In this study, a pathfinder network, which is abbreviated such that only important links are left for each node, was used as a graph-drawing algorithm for centrality and cohesion analysis.

#### Topic Modeling

The topic modeling method, which is a technique used in text mining, assumes that a document is a set of words and that each document contains multiple topics with a specific probability distribution [19-21,27]. For topic modeling, this study applied latent Dirichlet allocation (LDA), a classic type of machine learning method, to discover topic keywords that best represented the core value in the collection of documents [19,21,26]. In this study, a topic modeling analysis applying LDA was used to validate and provide evidence for a cohesion analysis which was the categorization of themes concluded by the authors. LDA was conducted using NetMiner 4.0. The values of the parameter settings were α=.01 and β=.01, and the number of iterations was 1000. The adequate distribution of topics was set at 7, showing a clear distinction between all topics. Probable keywords for each topic were chained to refer to the categorization results of the cohesion analysis.

## Results

### Characteristics of Included Research

In the last 5 years, an average of 335.4 articles per year were published on child and adolescent cancer survivors (SD 65.24). The number of studies was 252 in 2020, 429 in 2019, 303 in 2018, 349 in 2017, and 344 in 2016. The year in which the most number of child and adolescent cancer survivor–related articles were published was 2019, whereas the most recent year, 2020, had the lowest number of publications.

### Text Network Analysis

#### Degree Rank of 2-Mode (Word Documents), Degree, and Eigenvector Centrality of Keywords

Multimedia Appendix 1 presents the top 20–ranked keywords in the 2-mode network (word documents), degree centrality, and eigenvector centrality. Keywords that appeared most frequently in the documents included childhood cancer treatment (degree=925), risk (degree=748), effect (degree=681), diagnosis (degree=652), and cause (degree=578). In contrast, the top 5 keywords in terms of degree centrality were *risk*, *control interval*, *childhood cancer treatment*, *diagnosis*, and *radiation*. The degree centrality index was 26.606%, with a mean of 0.018 (SD 0.04). The index of the centrality ranges from 0% to 100%, indicating that the links were fairly distributed within the network when the percentage is closer to 0% [20]. The top 5 keywords in the eigenvector centrality were *risk*, *control interval*, *radiation*, *childhood cancer treatment*, and *respiratory rate*. The mean was 0.025 (SD 0.064. The trends in the theme of the keywords, in both degree and eigenvector centrality rank, revealed their similarity because 75% (15/20) of the keywords overlapped with each other.

#### Cohesion With Network Visualization

For cohesion analysis, the giant component was extracted from the 1-mode network, which represented the relationship between a set of keywords (main node vs main node) and documents (abstracts). The component included 90 keywords from the network consisting of link frequencies >60 based on the TF-IDF recoded value. A component community analysis, using the Blondel processing method, divided the groups into 3 clusters with a maximum modularity value of 0.258. Textbox 1 lists the clusters divided according to their connection strengths. Cluster 1 contains 34 keywords, including *risk*, *control interval*, *female*, *condition*, and *cancer center*. Cluster 2 contains 31 keywords, including *caregiver*, *effect*, *care*, *health*, and *group*. Cluster 3 contains 25 keywords, including *childhood cancer treatment*, *diagnosis*, *radiation*, *cause*, and *leukemia*. Multimedia Appendix 2 shows the top 10 keywords of each cluster and map of the communities.

**Results of cohesion (clusters; maximum modularity=0.258) and included keywords.**

**Cluster 1 (n=34): risk management**

- *Behavior, BMI, Cancer Center, cardiovascular Disease, Cohort, comorbidity, Comparison, Condition, Control Interval, Death, Deficiency, Diabetes, Engagement, Ethnicity, Exposure, Female, Gonadal, Hormone, Hospitalization, human papillomavirus, Insurance, Knowledge, Male, Malignancy, Mortality, Peer, Perception, Prognosis, Pulmonary, respiratory rate, Risk, Sex, Sibling, Surveillance*.

**Cluster 2 (n=31): health-related quality of life and supportive care**

- *Anxiety, Burden, Care, Caregiver, Central control station, Concern, Counseling, Depression, Education, Effect, Fatigue, Fertility, Group, Health, health care professional, Information, Intervention, Measurement, Nutrition, Physical activity, Physician, Posttraumatic growth, quality of life, Resilience, School, Stress, Support, Survivorship, Symptom, Transition, well-being*.

**Cluster 3 (n=25): cancer treatment and complication**

- *5 y, Assessment, BMD, Brain tumor, Cardiac, Cause, Central Nervous System, Chemotherapy, Childhood cancer treatment, Complication, Diagnose, Disease, Dysfunction, Experience, Incidence, leukemia, Lymphoma, magnetic resonance imaging, Medicine, Operation, Other cancer, Radiation, Regimen, Therapy, Transplant*.

### Topic Modeling

The keywords in the results of topic modeling, when applying LDA, feature the probability of being the best representation for the classified documents. In this study, 7 topics were determined to be appropriate matches in accordance with the results of the cohesion analysis. Multimedia Appendix 3 presents the keywords representing each topic based on a probability distribution with the number of documents included for each topic. Among the keywords representing the documents belonging to each topic, the first keyword has the highest probability of representing each topic, and the probability decreases with each keyword [28]. In this study, only the first and second keywords were considered for the analysis. Of the 1677 documents, topic 1 consisted of 265 (15.8%) documents, and the most probable keywords representing the documents belonging to this topic in order were *care* or *caregiver*. Topic 2 consisted of the lowest number of documents at 6.97% (117/1677), with *fertility* or *female* as representative keywords. Topic 3 had the largest number of documents at 21.36% (358/1677), with *care* or *caregiver* as the representative keyword. Topic 4 included 12.52% (210/1677) documents and was represented by the keywords *brain tumor* or *radiation*. Topic 5 consisted of 9.36% (157/1677) documents, with keywords including *physical activity* and *health*. Topic 6 consisted of 13.30% (223/1677) documents, with *risk* or *control interval* as representative keywords. Finally, Topic 7 contained the second largest number of documents at 20.69% (347/1677), with *complication* and *radiation* as representative keywords.

### Comparison of Results of Cohesion With Topic Modeling and Topic Naming

**Figure 3:**
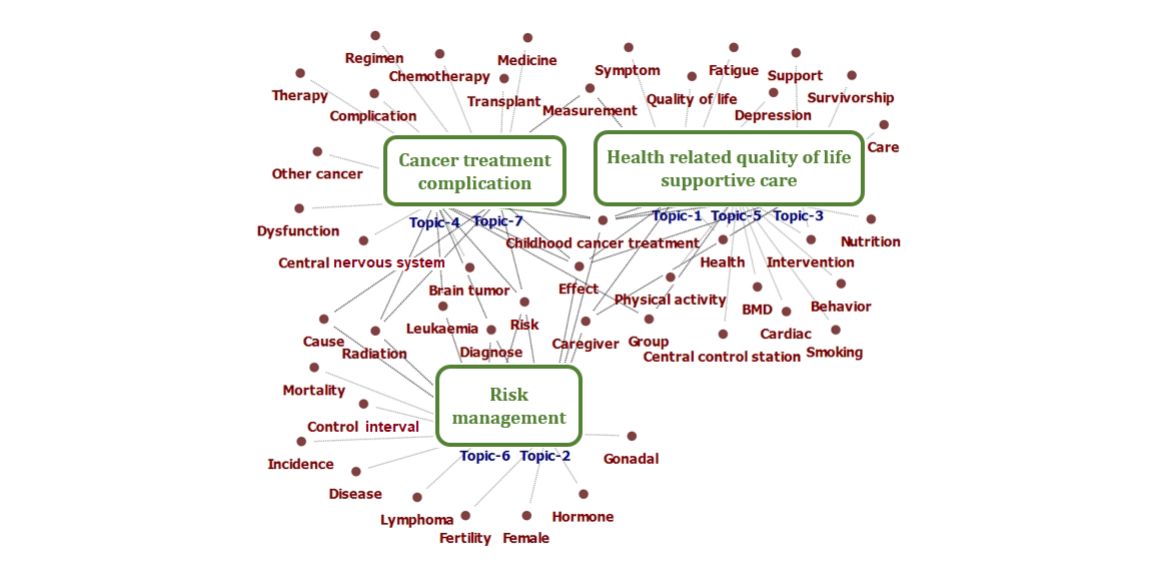
Comparison map of results between cohesion analysis and topic modeling.

On the basis of the keyword trends in each community, analyzed through cohesion, the authors reviewed the similarities among keywords, included in each cluster, in the results of the cohesion as well as each topic with representative keywords in the results of the topic modeling. As shown in Textbox 1 and Multimedia Appendix 3, comparing topics 2 and 6, we reached a consensus to name cluster 1 as *risk management*. Cluster 2 with topics 1, 3, and 5 was labeled *health-related QOL and supportive care*, and cluster 3 with topics 4 and 7 was labeled *cancer treatment and complication*. Figure 3 presents a clearer comparison of the keywords surrounding each categorized topic based on cohesion analysis and topic modeling.

## Discussion

### Principal Findings

In this study, child and adolescent cancer survivor–related research trends over the past 5 years were analyzed by reviewing papers published in all academic fields.

The main findings of this study were that the top 5 keywords in terms of degree and eigenvector centrality are risk, control interval, radiation, childhood cancer treatment, and diagnosis. Of 1677 documents, cluster 1 included 780 (46.51%) documents under *risk management*, cluster 2 contained 557 (33.21%) articles under *health-related QOL and supportive care*, and cluster 3 consisted of 340 (20.27%) studies under *cancer treatment* and *complications*.

Among the documents included in the 3 subgroups, the knowledge structure and research trends were described by comparing the results of the meta-analysis that presented research results regarding the concerned topics. The giant component from the centrality analysis in this study showed 3 subgroups based on the cohesion analysis. To name the cluster of subgroups more accurately, we compared the results of the cohesion analysis and topic modeling. Cohesion analysis has the advantage of showing the degree of cohesion between keywords belonging to the relevant community [17,28]. However, there is a limitation in that it does not have guidelines based on mathematical calculations to name a given community. Recently, topic modeling, in which keywords that can represent documents belonging to each topic are presented based on probability values, has frequently been used to supplement the limitations of cohesion analysis [20,21]. On the basis of keyword trends in each community with cohesion analysis, the authors reviewed the similarity among keywords included in each cluster in the results of cohesion analysis and each topic with representative keywords in the results of topic modeling. In addition, keywords representing each topic should be mutually exclusive [26]. In the topic modeling results of this study, the first and second keywords representing each topic did not overlap and were mutually exclusive. Therefore, the cohesion analysis and topic modeling results indicate that the clusters of keywords representing recent research trends were reasonably named.

In cluster 1, labeled *risk management*, keywords related to periodic risk management and various risks that child and adolescent cancer survivors could experience while living as survivors after cancer treatment were included. A total of 10 meta-analysis studies corresponding to cluster 1 were identified. In all, 6 meta-papers [29-34] regarding long-term risk and management needs were published, and smoking, binge drinking, and drug use were reported as vulnerable issues for child and adolescent cancer survivors. In particular, the importance of periodic surveillance for late risk management such as cardiac toxicity was reported in a study related to surveillance guidelines among 4 meta-papers [35-38] related to survivorship in child and adolescent cancer survivors. On the basis of this analysis, it is necessary to conduct various studies related to education, information provisioning, health care–provider training, and social support and care system establishment related to periodic surveillance systems for potential risk management.

In cluster 2, labeled “health-related QOL and supportive care,” keywords related to the various experiences of child and adolescent cancer survivors’ care, and QOL required for survivorship were included. A total of 30 articles were identified in the meta-analysis studies corresponding to cluster 2. Most meta-studies on the development and effect of interventions related to physical health behaviors, such as exercise, nutrition, health promotion behavior, and obesity prevention, were found in 13 articles [39-51]. The results of this study related to keywords such as *effect*, *health*, *physical activity*, *intervention*, and *nutrition* were also confirmed. Specifically, studies proposed that future research should focus on providing evidence of the efficiency and feasibility of interventions that use web-based technologies (such as @TheTable e-cookbook) to facilitate remote intervention delivery and peer support as the most effective means of promoting changes in health behavior [39-41,49,50,52]. We identified *anxiety*, *burden*, *depression*, *resilience*, *stress*, *counseling*, *measurement*, and *post-traumatic growth*. In 7 meta-analyses [53-59], child and adolescent cancer survivors were highly likely to experience mood or affective disorders [56], and brain tumor in child and adolescent cancer survivors was significantly associated with anxiety, depression, and health-related QOL, highlighting the importance of psychosocial screening. Furthermore, in 1 systematic review and meta-study [54], dealing with the keyword *post-traumatic growth* identified in this study, the need for targeted social support, clinical intervention, and education to facilitate posttraumatic growth was suggested through an analysis of 18 studies. Other keywords related to academics, school, and social adaptation were *education*, *group*, *school*, and *transition*, with 6 meta-studies [60-65] related to these keywords. In particular, the results of 26 studies were analyzed in a meta-study conducted by Saatci et al [65], and compared with controls there were significant differences in educational attainment among survivors according to country and culture. It has been reported that the support and participation of a cross-professional group, such as that of clinicians, teachers, and policymakers, is needed, and the problem of academic and school adjustment for child and adolescent cancer survivors involves an extremely critical developmental issue and an important global care problem that should not be overlooked. A total of 3 studies [66-68] addressed the fertility issues among child and adolescent cancer survivors. The most commonly reported barriers were a lack of patient educational materials regarding oncofertility psychosocial support and staff training [67], whereas another study attempted to realize a qualitative meta-synthesis [69] by synthesizing 51 research results into 5 themes (ie, experiencing difficulties related to cancer, fluctuating realities, coping strategies, new roles and responsibilities of the child, and practical resources to enable the managing of cancer). Compared with the keyword-analysis results confirmed for cluster 2 of this study, it is necessary to continuously conduct research on adaptation and improvement of QOL for children with cancer in terms of their physical, psychological, social, future occupational, and reproductive problems.

Cluster 3 of the cohesive analysis was labeled *cancer treatment and complication* because the main keywords were matched with topics 4 and 7 of the topic modeling results. Among the relevant papers, 11 meta-analysis studies [48,70-79] were identified, and the correlation with keywords included in cluster 3 of this study was confirmed. Keywords related to major carcinomas were *leukemia*, *brain tumor*, and *lymphoma*, and for other cancer types, *other cancer* were designated as representative words. Words related to *childhood cancer treatment* and *diagnosis* were the main keywords. Meta-studies were conducted on the subject of complications and therapeutic management of the endocrine system [78], nervous system [71,77,79], heart [48,72-74], tinnitus [75], and infection [76]. In particular, meta-analyses related to cardiac toxicity were predominant in 4 articles [48,72-74]. Most of the studies conducted in the medical field were identified as the main characteristics of the studies that fall into cluster 3, and the characteristics of interdisciplinary research topic keywords were confirmed.

### Implications

The purpose of this study was to identify the knowledge structure based on major research trends and core research topics related to all child and adolescent cancer survivors. The 3 categories (*risk management*, *health-related QOL and supportive care*, and *cancer treatment* and *complications*) imply the current research trends. The results of this study are significant in that they can suggest practical research directions for the specific needs regarding QOL of child and adolescent cancer survivors in all academic fields.

In terms of the significance of the research methodology, this is the first study to use a text network analysis on child and adolescent cancer survivor–related topics and categorize interdisciplinary research trends. It has the advantage of increasing the validity of research trends as a quantitative method, based on a higher probability, by comparing a cohesion analysis with topic modeling.

### Limitations

This study included data from only the last 5 years. The limitation is that the trends in child and adolescent cancer survivor–related research conducted based on the past 5- or 10-year periods cannot be compared or analyzed. The results of this study, focusing on the meta-analysis results included in each cluster, were analyzed and compared with 1677 studies in this research area. Therefore, potential limitations could exist, as some topics have been addressed infrequently in some studies; therefore, these might have been overlooked. Finally, studies in the genetic and biochemical fields were excluded.

### Conclusions

This study is significant, in that it confirmed the knowledge structure based on the main keywords and cross-disciplinary research trends related to child and adolescent cancer survivors published in the last 5 years worldwide. To more accurately name the knowledge structure on a quantitative basis, the results of coherence analysis and topic modeling were compared and analyzed based on the giant component confirmed through a centrality analysis. In both cluster 1, *risk management*, and cluster 2, *health-related QOL and supportive care*, many research results for multidisciplinary teams have also been published.

Regarding cluster 3—*cancer treatment and complications*—papers published in the medical field prevailed, confirming the identity of interdisciplinary research topics. Improving QOL is the primary goal of child and adolescent cancer survivors, preventing and managing various aspects of problems encountered during the transition to normal life. To this end, it is necessary to further revitalize the studies through a multidisciplinary team approach to promote age-specific health behaviors and develop intervention strategies with increased feasibility and educational effects for child and adolescent cancer survivors.

## Appendix

Multimedia Appendix 1 Top 20 keywords by degree, degree centrality, and eigenvector centrality.

Multimedia Appendix 2 Top keywords and maps of clusters.

Multimedia Appendix 3 Results of topic modeling (using latent Dirichlet allocation; N=1677).

## Abbreviations

LDA: latent Dirichlet allocation
PRISMA: Preferred Reporting Items for Systematic Reviews and Meta-Analyses
QOL: quality of life
TF-IDF: term frequency-inverse document frequency
