# Supplementary figures and images for: Web-Based Research Trends on Child and Adolescent Cancer Survivors Over the Last 5 Years: Text Network Analysis and Topic Modeling Study
Source: J Med Internet Res. 2022 Feb 1;24(2):e32309. doi: 10.2196/32309 (PMC8848247; doi:10.2196/32309)

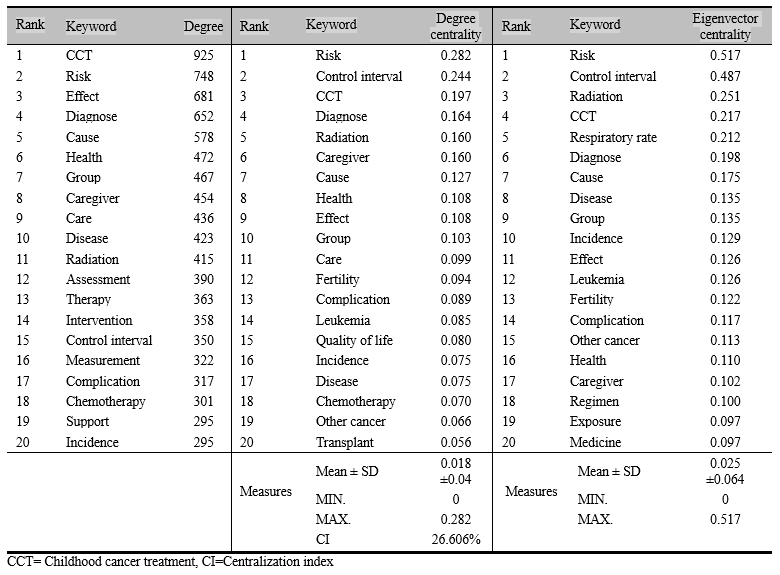

Supplement: Multimedia Appendix 1 [file jmir_v24i2e32309_app1.png]

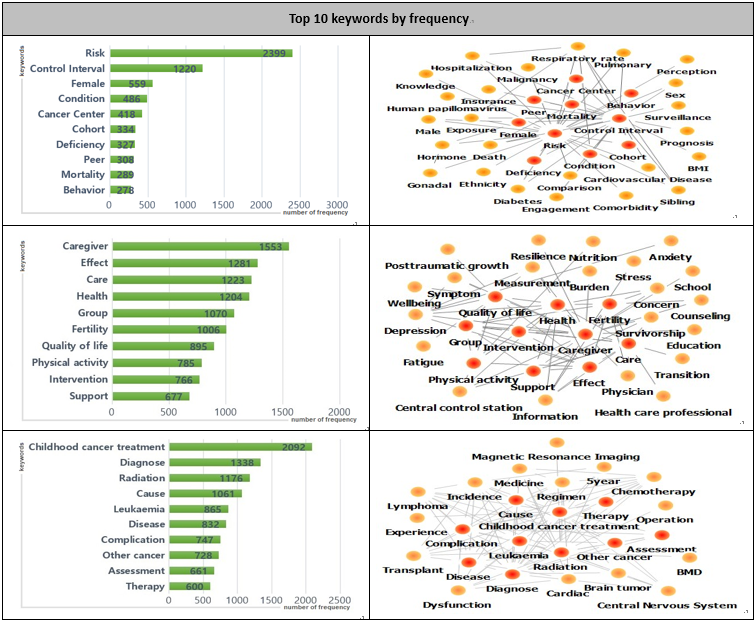

Supplement: Multimedia Appendix 2 [file jmir_v24i2e32309_app2.png]

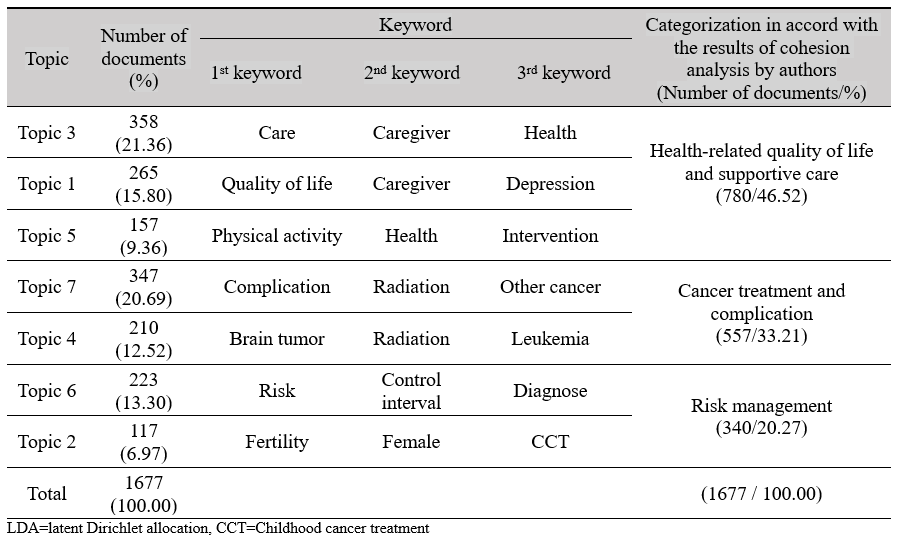

Supplement: Multimedia Appendix 3 [file jmir_v24i2e32309_app3.png]
